# Molecular origin of drug release by water boiling inside carbon nanotubes from reactive molecular dynamics simulation and DFT perspectives

**DOI:** 10.1038/s41598-017-04981-2

**Published:** 2017-07-05

**Authors:** M. Darvish Ganji, Sh. Mirzaei, Z. Dalirandeh

**Affiliations:** 1 Department of Nanochemistry, Faculty of Pharmaceutical Chemistry, Pharmaceutical Sciences Branch, Islamic Azad University (IAUPS), Tehran, Iran; 2 Young Researchers & Elite Club, Pharmaceutical Sciences Branch, Islamic Azad University, Tehran, Iran; 3 Young Researchers and Elite Club, Central Tehran Branch, Islamic Azad University, Tehran, Iran

## Abstract

Owing to their nanosized hollow cylindrical structure, CNTs hold the promise to be utilized as desired materials for encapsulating molecules which demonstrate wide inferences in drug delivery. Here we evaluate the possibility of drug release from the CNTs with various types and edge chemistry by reactive MD simulation to explain the scientifically reliable relations for proposed process. It was shown that heating of CNTs (up to 750 K) cannot be used for release of incorporated drug (phenylalanine) into water and even carbonated water solvent with very low boiling temperature. This is due to the strong physisorption (π-stacking interaction) between the aromatic of encapsulated drug and CNT sidewall which causes the drug to bind the nanotube sidewall. We have further investigated the interaction nature and release mechanism of water and drug confined/released within/from the CNTs by DFT calculations and the results confirmed our MD simulation findings. The accuracy of DFT method was also validated against the experimental and theoretical values at MP2/CCSD level. Therefore, we find that boiling of water/carbonated water confined within the CNTs could not be a suitable technique for efficient drug release. Our atomistic simulations provide a well-grounded understanding for the release of drug molecules confined within CNTs.

## Introduction

Nanotechnology has been devoted much attention as a superior tools for drug delivery system to overcome typical formulations in the past decades^1, 2^ and is in progress for use inside the human body^3^. Manufacturing the drug delivery system to the target area is a crucial medical issue. Penetrating through the cell membrane often creates serious problems. In particular, polar drug molecules prefer to stay in the hydrated environment and avoid hydrophobic double lipid layer environment. In this regard, the transmission systems provide special form mechanism of drug release, which is interesting in terms of application, since they have a large effectiveness in medical treatment^4^.

Since the discovery of carbon nanotubes (CNTs) in 1991^5^ and earlier in 1952^6^, CNTs have come to the focus of attention, and their benefits can be currently found^3^. The idea of using a hollow cylindrical structure as a transporter or container has been proposed for many years. Most recently, special properties of these novel materials were found in the medical communities with the hope to develop a new drug delivery system for small treatment agents such as proteins, nucleic acids, and other biologically active molecules^6–9^. CNTs are used due to their unique properties in drug delivery^9^, sensors as drug delivery agents^10, 11^, destruction of tumors with increasing temperature^12^, cholesterol and blood sugar measurements^12, 13^, identification of antigens and DNA bonding with no toxic effects^5, 10, 11, 14–17^. Given the potential abilities of CNTs inside the cells, the potential application of CNTs is used for transport and delivery of drugs and antibodies without toxic effects^18–24^. Their reduced toxicity effects are perhaps due to their small size, stable structures and surprising nature. It is absolutely due to their special surface, they can be attracted to a variety of therapeutic molecules^25^ or bond to them. Generally, CNTs are used to treat diseases through binding with therapeutic molecules and influencing the target cells^3^.

A number of researchers used the simple non-covalent system for binding of reactant molecules to CNTs walls^26, 27^. Their needle shape helps them to reach the target cells through penetrating into the cell membrane and transporting of therapeutic molecules^28^, and others showed that the CNTs are ideal probes^27^. More recently, it was shown that CNTs easily penetrate into the biological cells^29^, and make them functionalized through covalent and non-covalent bonds; thus, their biological availability becomes more effective^30–33^ and their toxicity effects would reduce^29, 34^; these properties are in favor of the new transition systems. CNTs, on the other hand, are active in the field of optical spectrum; therefore, they are transparent for biological tissues and can provide excellent features for the delivery of therapeutic agents with the help of optical control^35–37^.

In recent years, many efforts have been done for the advancement of mechanisms related to drug release through CNTs^4^. For example, some proteins are encapsulated within the space of CNTs^15, 38^, or attached to the walls through non-covalent bonds^39, 40^. In addition, the proteins attaching to the lateral walls of CNTs were investigated, and the adsorption of these molecules on the surface of CNTs were considered^41, 42^. On the one hand, the nanosyringes subject was introduced^43, 44^. To this end, a piston should be embedded inside the nanotube and ferromagnetism^45^ and CNTs with small diameters^46^ were needed. However, this nanodevice is actually complex and has not yet been fabricated in practice. Few clinical methods for screening molecules and attaching to them and also target the cancer cells have been already approved^47^. The reasons include unclear details of these interactions due to laboratory constraints^48^, high complexity of such devices and non-developed methods^4^. However, simulations showed that optical heat of the CNTs for encapsulated drug delivery can be used, and valuable details of the process can be provided. Molecular simulation is one of the advantage methods to directly investigate atomic adsorption details through molecular surface dynamics. Also, surface chemistry effects on the adsorption behavior of proteins can be discussed^3^.

Meanwhile, water trapped in the nano**-**space plays an important role in chemical reactions involving the solid**-**liquid interface. Water passes through cellular membranes and controls the biochemical activities; thus, understanding the structure and behavior of water in the nano**-**space is essential. In its liquid state, water molecules form hydrogen bonds with water molecules surrounding and produce a complex structure^49–51^. In addition, the weak interaction among water and the hydrophobic wall of CNTs minimizes the friction in a narrow nanotubes^52^. The interfacial friction of water at carbon nanotubes interfaces (inside and outside of CNT) with various curvatures of nanotubes has been studied by both equilibrium and non-equilibrium molecular dynamics (MD) simulations^52^. The results indicated that the friction coefficient strongly depends on the nanotubes curvature and decreases for confined water inside the tube with decreasing the CNT diameter^52^. Their MD simulation results support the experimental reports on the fast transport of water in nano-sized CNT based membranes. Also, the properties and flow rate into CNTs with a small diameter were compared with natural biological channels^53^, and demonstrated that the flow into CNTs provide better results than the performance of biological water channels^54^. Hammer *et al*. showed osmotic transfer of water inside the CNTs using MD simulations^55, 56^. Holt *et al*. demonstrated experimentally that water transport through CNTs membranes is fast^57^. Chaban *et al*. also examined the dependence of water transfer and water vapor pressure trapped inside the CNTs to the diameter of CNTs and temperature^58–60^. They performed MD simulations of water droplets confined within the armchair CNTs with various diameter over a wide range of temperatures. Their simulation results showed that while water droplets under confinement generate little vapor pressure however disintegrate at a lower temperature than in the bulk phase. This different behaviors of the free and confined water droplets in the evaporation mechanisms has been rationalized by exploring the interaction nature between CNT and water droplet. They reported that confined water exhibit sharp increase of pressure inside the nanotubes upon heating and claimed that their scheme could be employed in therapeutic applications of CNTs^60^. Recently, Pérez-Hernández and co-worker systematically investigated the anisotropy effects on the structure and dynamics of water molecules confined within low-diameter CNTs by MD simulation at room temperature^61^. They found that water inside (6, 6) CNT forms frequent chain ruptures by considering anisotropic of the water–carbon interaction while one-dimensional ordered chains was formed in isotropic model. In the case of (7, 7) CNTs the structures of confined water demonstrate a non-monotonous dependence on the anisotropy potentials. However, for the (8, 8) CNTs the structure and dynamics of water were found to be in different situation and almost independent of the anisotropy potential. This can be attributed to the higher stability of water molecules which form non-helical fivefold prisms since the water–water interaction prevails over that of the water–carbon interaction. They conducted that this behaviour might be extended to the larger diameter CNTs. As a rule of thumb, water–carbon and water–water interactions play an important role in the structure and dynamics properties of confined water within CNTs. In other research works, both cluster and single-layer structures have been observed in the transfer and release processes of water molecules^62–64^. In addition, hydrogen bonds have non**-**understandable structures; therefore, the mechanism of release and transport of water remains unknown^65^.

A number of simulations were performed for carbon nanotube-based drug delivery and drug delivery system based on CNTs by classical MD simulation^43, 46^. For example^46^, peptide confined within nanotubes is done by competitive replacement process, and releasing occurs through van der Waals (vdW) interactions. In a novel work, Chaban *et al*. investigated dependence of the boiling process on CNT diameter by using classical MD simulation^58^. They showed the strong dependence of the boiling process on CNT diameter and suggested that infrared radiation energy might be employed to reach precise control over the release of incorporated drug. Indeed, fast increasing of pressure inside the CNTs at temperatures above the boiling point as well as vital dependence of boiling temperature on diameter advocate a novel drug release proposal. According to their suggestion, the high pressure generated by evaporated liquid can pushes drug molecules outside. Despite their novel suggestion, the release of incorporated drug into CNTs has not yet been evaluated and hence this important phenomenon needs to a systematic study to explain the specific molecular factors for proposed drug release mechanism.

The main objective of this work is therefore to assess the unique properties of CNT for drug release by using the state-of-the-art reactive MD simulations, to approve or refute the suggested proposal. We further give scientific insight into the detailed water uptake/release in/from CNTs accompanied by drug release. To this end, we have performed comprehensive calculations on the interaction of water/drug with inner side wall of CNTs, using MD simulations as well as first-principles calculations. Altogether, our results do indeed confirm that CNTs act as fast water transporters, pointing to an unforeseen mechanism at the origin of the ultralow water-carbon friction. We employ MD simulation coupled with reactive force field ReaxFF which is a bond-order-dependent potential and describes accurately bond forming and breaking for molecular systems. The key feature of ReaxFF is consideration of polarization effects which play an important role in interacting systems. The validity of the ReaxFF potential has been evaluated by quantum mechanical calculations at the DFT level of theory. We selected different types of CNTs consisting of armchair, zigzag and chiral nanotubes with diameters and lengths of about 2.0 and 3.0 nm, respectively, to predict the release properties of confined molecules inside the nanotubes. Phenylalanine (Phen) amino acid is considered as a suitable model of drug which consists of both polar side and aromatic ring. Various solvents have been considered from pure water to binary mixture involving water and carbon dioxide with different percent which causes too low boiling temperatures. Furthermore, the effect of CNT edge on the drug release was also examined. Our reactive MD simulations which performed at temperatures ranging from 300 to 700 K reject the idea that boiling of water confined inside the CNTs causes the encapsulated drug to be released. It is expected that these findings could provide fruitful information for efficient drug release in encapsulated biomolecules inside the nanotubes.

## Simulation Procedure

The MD simulations were carried out with the “General Utility Lattice Program” (GULP)^66^ which is designed to perform a variety of tasks based on force field methods. It is a flexible tool for the simulation of nanostructured based materials through interatomic potential models^66^. One of the most important features of GULP is the implementation of reactive force**-**field which involves the formation and breaking of chemical bonds. Conventionally, researchers modeled interactions between atoms using molecular dynamics with fixed-bond topology force fields such as AMBER, OPLS, CHARMM, and COMPASS to model covalent systems. In these molecular modeling methods, bonds are defined at the start of a simulation and remain unchanged throughout the simulation procedure. However, since bond forming and breaking play an important role in some molecular systems, a new class of force fields, such as the recently developed Reax Force Field^67^ (ReaxFF), are necessary to simulate the respective systems. Further, ReaxFF can handle the charge transfer between atoms through the EEM charge equilibration method^67^ which is important in interacting systems. The use of ReaxFF parameter sets expands to scope of materials research and have been demonstrated to precisely describe bond breaking and forming for various carbon-based systems^68–76^, oxidation of hydrocarbons^77^, and catalytic formation of nanotubes^78, 79^, to name just a few.

**Figure 1:**
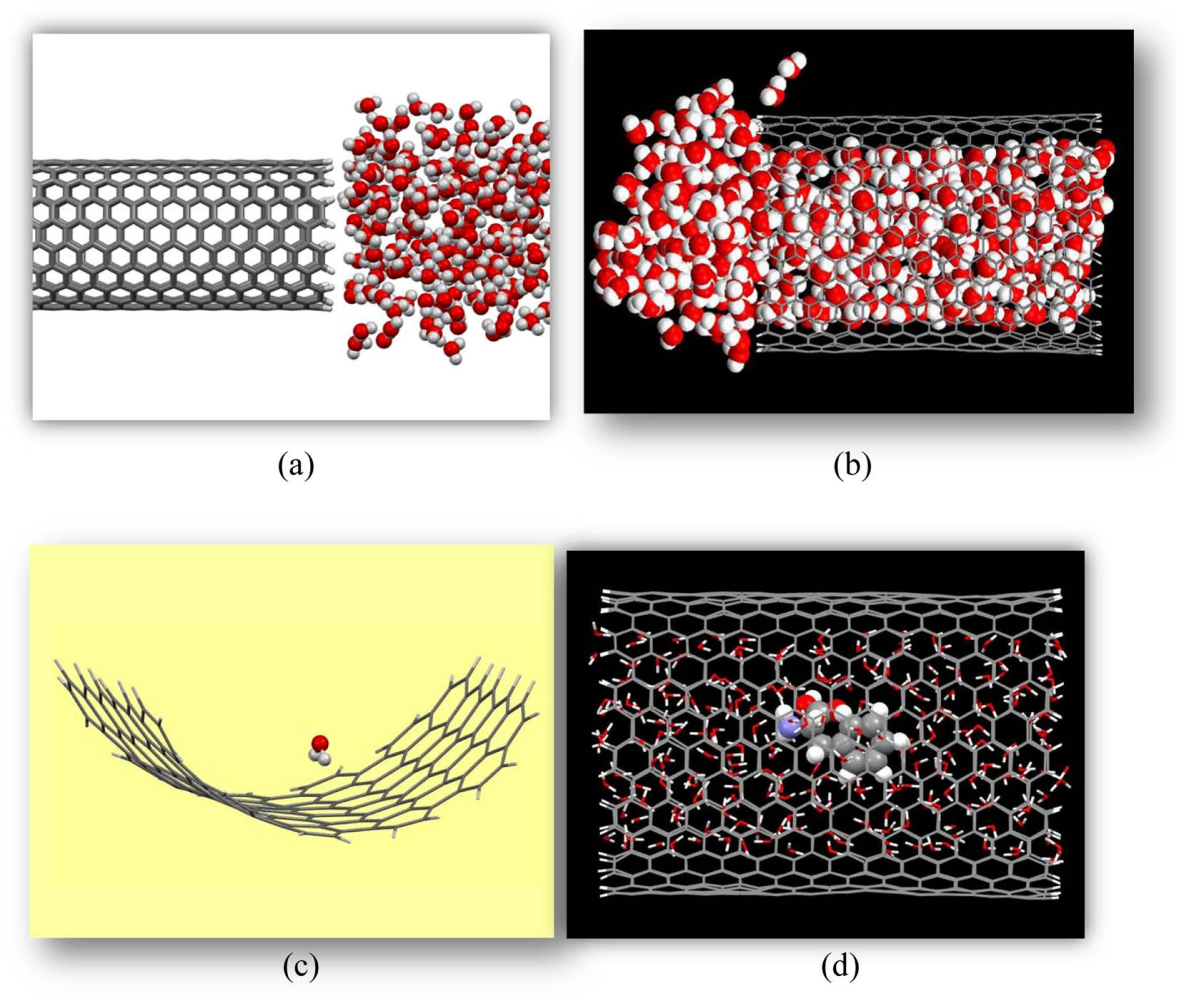
Schematic representation of water/CNT system (**a**) before and (**b**) after optimization within MD simulation, (**c**) schematic representation of a single water molecule attached to the interior sidewall of a (15, 15) CNT, (**d**) water molecules and phenylalanine as a drug molecule confined in a (26, 0) CNT.

We used a 0.25 fs time**-**step and the leap-frog integration algorithm for solving the Newton’s equations of motion. The several steps have been considered in the simulation procedure. First, we set an empty single-walled CNT (SWCNT) in the center of simulation box accompanied with liquid water beside the nanotube as depicted in Fig. 1. It should be noted that, no water molecules were placed inside the CNT and on its exterior surface. The NVT ensemble was utilized with a Nosé−Hoover thermostat and temperature was maintained at 300 K with an initial Gaussian velocity distribution consistent with this temperature. We then carried out 100 ps of simulation times in order to obtain the optimized structures of the systems under study while selected CNTs were completely filled with water at room temperature (see Fig. 1). We then removed all water molecules that still remained outside the nanotube and a phenylalanine (Phen) amino acid was embedded into the water molecules. The resulting system was additionally equilibrated in the constant temperature and constant volume during 100 ps. Finally, each MD system was gradually heated from 300 to 700 K during 2000 ps, involving 310 K which corresponds to the human body temperature. The temperature of the system was gradually increased at a rate of approximately 0.1 K per picosecond.

All DFT calculations including structural optimization and total energy calculations were performed using the ORCA suite of programs^80^ (version 3.0.0^81^. The geometries of the investigated molecules were optimized at the revPBE^82^/def2-SVP^83, 84^ level of theory. The total energy calculations for interaction energies estimation were performed with the TZVP(-f) basis set. The atom-pairwise dispersion correction (D3^85, 86^) with Becke–Johnson damping (D3(BJ))^87^ was also utilized for all systems. We also used the well-tested density-fitting (resolution-of-the-identity approximation) and chain-of-sphere methods (RIJCOSX)^88^ to accelerate the calculations at a reasonable accuracy. We applied tight self-consistent field (SCF) convergence criteria and large possible integration grid (Grid5) to achieve accurate energies on the minimum energy pathways by fully converged stationary points.

## Results and Discussion

For clarity purposes, we divide this section into five subsections. The first section deal with the water uptake by various CNTs with MD simulation. The second three subsections respectively concern with the release of incorporated drug into water solvent, carbonated-water solvent and mechanism of the release of drug and water molecules from the CNT. Moreover, the effect of CNT edge on the drug release will also be explored. These three subsections take advantage of hiring both reactive MD simulations and DFT calculations. Last subsection of the current work will be dedicated to evaluate the quality of our computational approaches throughout this study where the accuracy of the obtained results will be evaluated by some experimental values as well as high level quantum mechanics benchmark methods.

### Water uptake by various CNTs

We first systematically investigate water uptake by various SWCNTs under capillary action. The distribution of water molecules inside CNTs are examined using equilibrium MD simulation in a NVT ensemble at room temperature (298 K). We have computed the filling velocities of water molecules inside various types of SWCNTs viz. zigzag (26, 0), chiral (21, 9) and armchair (15, 15) CNTs as represented in Fig. S1, corresponding to diameters of 19.73, 22.09, and 20.27 Å, respectively. We have measured the number of water molecules passing through the SWCNTs as a function of time. The lengths of selected SWCNTs were chosen to be about 30 Å (3.0 *n*m). The number of water molecules transported through the above mentioned CNTs associated with the details of their filling rates is illustrated in Table 1. Further, the filling time and volumetric flow rate into each CNT are given in Table 2. Considering the above mentioned SWCNTs having the same length and almost the same diameter we find that armchair CNT was filled in the shortest time while the number of water molecules confined within CNTs was the highest for chiral CNT. Therefore, the capillary theory agree very well for the (15, 15) CNT. Because of this, we can conclude that armchair CNTs exhibit better adhesion at room temperature between water molecules and its interior sidewall than the other counterparts. The schematic representation of water molecules incorporated into the (15, 15) CNT is shown in Fig. 1.

**Table 1:** The number of enclosed water molecules (*N* water) in different time intervals for various CNTs.

Time (ps)	*N*_H2O_: (15, 15) CNT	*N*_H2O_: (26, 0) CNT	*N*_H2O_: (21, 9) CNT
10	37	20	36
20	45	34	80
30	78	59	82
40	98	72	104
50	130	79	139
60	161	104	182
70	214	118	211
80	**236**	138	258
90	—	160	**289**
100	—	175	—
110	—	201	—
120	—	**222**	—

**Table 2:** The filling time and volumetric flow rate into considered CNTs.

CNT	*t* (ps)	Q (*m* ^2^/*s*)
(15, 15)	65	1.39 × 10^−16^
(21, 9)	73	1.60 × 10^−16^
(26, 0)	114	7.78 × 10^−17^

We now evaluate the adhesion nature between confined water molecules and CNTs sidewall by performing single point calculation with ReaxFF potential for optimized nH_2_O@CNTs system through MD simulation. For this end, the interaction energy between confined water molecules within (15, 15) CNT and (26, 0) CNT and interior sidewall of the tube was calculated by following relation:

1 $${E}_{{\rm{int}}}=E({{\rm{nH}}}_{2}{\rm{O}}@{\rm{CNT}})-[E({{\rm{nH}}}_{2}{\rm{O}})+E({\rm{CNT}})]$$

where *E*(nH_2_O@CNT) indicates the total energy of nH_2_O confined in CNT. *E* (nH_2_O) and *E* (CNT) describe the total energies of water molecules and pristine CNT, respectively. The calculated interaction energies for 236H_2_O@CNT (15, 15) and 222H_2_O@CNT (26, 0) systems are estimated to be respectively −14.58 eV (−336.13 kcal/mol) and −13.02 eV (−300.22 kcal/mol). It was clearly found that water molecules tend to bound stronger to the interior sidewall of armchair (15, 15) CNT rather than zigzag (26, 0) counterpart at room temperature. This finding confirms our MD simulation result of better adhesion between water molecules and interior sidewall of (15, 15) CNT which leads to a faster water uptake by armchair nanotube.

To validate the accuracy of classical ReaxFF potential in interaction energy estimation we have further carried out complementary calculations with the *ab initio* DFT method. Due to computational limitations with the DFT method, the interaction between a single H_2_O molecule and interior sidewall of CNT was investigated. Furthermore, since the consideration of the whole system of CNT is impossible thus we have selected a piece of nanotube through cluster modelling (see Fig. 1) which has been shown to be suitable model for such systems^89–92^. The calculated interaction energy with the DFT-revPBE-D3 method was found to be −0.24 eV and the obtained value by the ReaxFF potential was estimated to be −0.23 eV. This reasonable agreement between two computational approaches assure that our employed ReaxFF potential gives reasonable results for systems under study.

### Release of incorporated phenylalanine into water solvent

As previously mentioned, it was proposed that thermal energy delivered to the CNT by a laser light can be used to accelerate the motion of the molecule inside the tube and hence its release from the nanotubes^58^. It can also be used to weaken and disrupt the CNT**-**molecule interaction. The local temperature around the nanotubes is controlled by adjusting the laser radiation intensity that results in preventing the death of target biological cell.

Here, we investigate the effect of CNT heating on the behavior of water molecules and phenylalanine confined inside the aforementioned CNTs (Phen/water@CNT system) (see Fig. 1). To this aim the MD simulations were performed at temperatures ranging from 300 to 700 K for the Phen/water@CNT systems. Our reactive MD simulations results don’t support the idea that heating of CNTs causes the encapsulated drug molecule to be released. However, it is necessary to note that release temperature should be higher than the temperature of the human body, since otherwise the encapsulated drug molecule will leave the CNT before it is delivered to the target tissue. Although the temperature increases from 305 to 355 K doesn’t significantly influence the effectiveness of the drug release, the phenylalanine molecule somewhat moves longitudinally and laterally with the transport of water molecules. Given the cross distance between the phenylalanine and the inner sidewall of the tube, one can explain that perhaps there is a strong interaction between the CNT and phenylalanine avoiding its release in this temperature range. For instance, the position details of the phenylalanine molecule incorporated into the CNTs at 50 ps of simulation time is given in Table S1.

**Figure 2:**
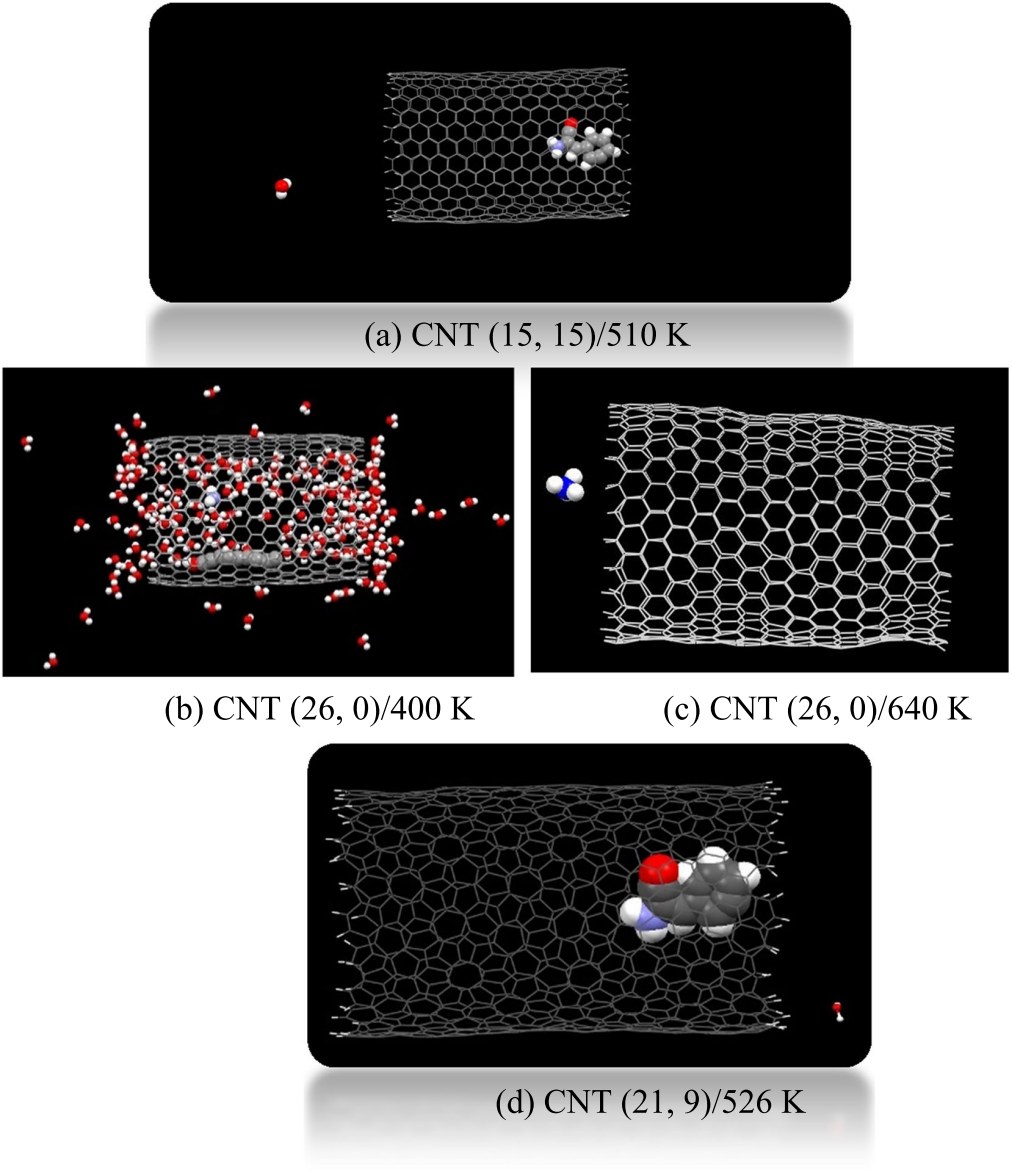
The release process of molecules confined in (**a**) CNT (15, 15) at the temperature of 510 K, (**b**) CNT (26, 0) at temperature 400 K, (**c**) CNT (26, 0) at temperature 640 K and (**d**) CNT (21, 9) at temperature 526 K.

We found that water molecules completely get released from the (15, 15) CNT at 510 K (see Fig. 2). Release process of water droplets from CNTs can be explained as follows. At low temperatures, water molecules with high kinetic energy escape the droplet but are immediately adsorbed on CNT walls due to CNT-water attraction. As the system is heated, the kinetic energy of water rises and consequently water begins to boil and then results in liquid/vapor phase transition, which works to overcome the van der Waals attraction between water molecules and CNT side walls and so cause to rise the inside pressure and hence release of water molecules from the nanotube. Furthermore, its distance from one end of the nanotube during the simulation process was equal to about 28 Å therefore, phenylalanine has reached to the end of the nanotube but has not exited yet. Our simulation results show also that the increase of temperature up to 670 K doesn’t cause the phenylalanine to leave the nanotube, but it results in the phenylalanine to be treated to start decomposition with removal of its amino group. Moreover, the nanotube wall slightly deforms due to interactions between molecules confined inside the tube and its inner sidewall. The encapsulated drug molecule remains inside the CNT, probably as a result of π-stacking interactions between the phenylalanine and the nanotube sidewall. The average equilibrium distance between the aromatics ring of phenylalanine from the nanotube surface is determined to be about 3 Å. It was also found that the encapsulated drug was decomposed when the temperature inside the zigzag (26, 0) CNT reached to 400 K and an ammonium ion was formed, as shown in Fig. 2. By increasing temperature to about 500 K, water molecules completely get released from the (26, 0) CNT and only the ammonium ion remains inside the tube which exits the nanotube at about 640 K, as represented in Fig. 2. Because of the surviving interactions and temperature increase, the nanotube ends diameters changed to 20.399 and 27.958 Å at this stage. As shown in Fig. 2, water molecules completely get released from the (21, 9) CNT at 526 K, while the phenylalanine remains inside the nanotube which can be attributed to the strong interaction between the phenylalanine and CNT sidewall. It is interesting to note that similar trends have also been observed for the system containing the (21, 9) CNT, phenylalanine and water molecules. The average equilibrium distance of the encapsulated molecule from the sidewall of the (21, 9) CNT was calculated to be about 3 Å. We found that the release of encapsulated drug molecule inside the (15, 15), (26, 0), and (21, 9) CNTs was happened at the temperature of about 720, 695, and 560 K, corresponding to 420, 370, and 259 ps, respectively. It should be noted that these temperatures are remarkably higher than the human body temperature. Present simulation results reveal that heating of CNTs in temperatures ranging from 305 to 355 K cannot be used for release of the encapsulated phenylalanine from the SWCNTs. Furthermore, raising temperature more than 500 K not only didn’t cause the molecule to release from the CNTs, but also treated the confined molecule to start decomposition inside the zigzag (26, 0) CNT.

### Release of incorporated phenylalanine into carbonated water solvent

Up to now, we found that pure water solvent could not release the encapsulated drug molecule from the nanotubes. Hence, we examine binary mixture involving water and carbon dioxide which is known as carbonated water. The use of carbonated water offers the advantage of well**-**controlled drug release within the ideal temperature range because of the too low boiling temperature of CO_2_ in comparison with the pure water. Indeed, upon modest heating of CNTs, binary mixture of water and CO_2_ confined inside the CNTs will release CO_2_ molecules and generate high pressure which might help to facilitate drug release from the nanotube at the temperature of the living body. Here, we evaluate the drug release of systems containing the CNTs, phenylalanine and binary mixture of water and CO_2_ corresponding to temperatures ranging from 300 to 350 K. For this aim we consider different CO_2_ percentages of 20, 40, and 55% in the mixture.

Similar MD simulation procedure were carried out for the considered systems. Our atomistic simulation results show that under the above mentioned simulation conditions, the encapsulated phenylalanine didn’t leave the CNTs and the solvent molecules did not completely exit the nanotube (see Fig. S2). This might be likely because of the strong interactions between the solvent molecules and the inner sidewall of these nanotubes. When the binary mixture was involving 20% of CO_2_ the drug molecule was decomposed in the (26, 0) CNT due to the acidic media in the nanotube. Increasing the CO_2_ percentage to 40% causes the solvent to become more acidic which results in decomposition of the drug molecule inside the (26, 0) CNT and also (15, 15) CNT. When the CO_2_ percentage was raised to 55%, the drug molecule was decomposed inside the (21, 9) and (26, 0) CNTs, while the molecular structure of confined phenylalanine within the (15, 15) CNT remain unchanged upon the temperature raising. The position details of the phenylalanine molecule incorporated into the considered systems is tabulated in Table S2. Release phenomena of the encapsulated drug inside the (15, 15), (26, 0), and (21, 9) CNTs filled by binary mixtures of water**-**CO_2_ was also examined assuming that the temperatures of these systems raised higher than the human body temperature. As can be seen, similar to the previous results obtained in temperatures ranging from 300 to 350 K, the encapsulated drug could not get released from the CNTs and the solvent molecules did not completely leave the nanotube. For example, heating of (15, 15) CNT up to 509 K did not cause the confined solvent molecules to exit completely from the nanotube. From the comparison of these result with those of pure water systems one can conclude that presence of CO_2_ molecules prevent the solvent molecules to leave completely from the nanotube.

**Figure 3:**
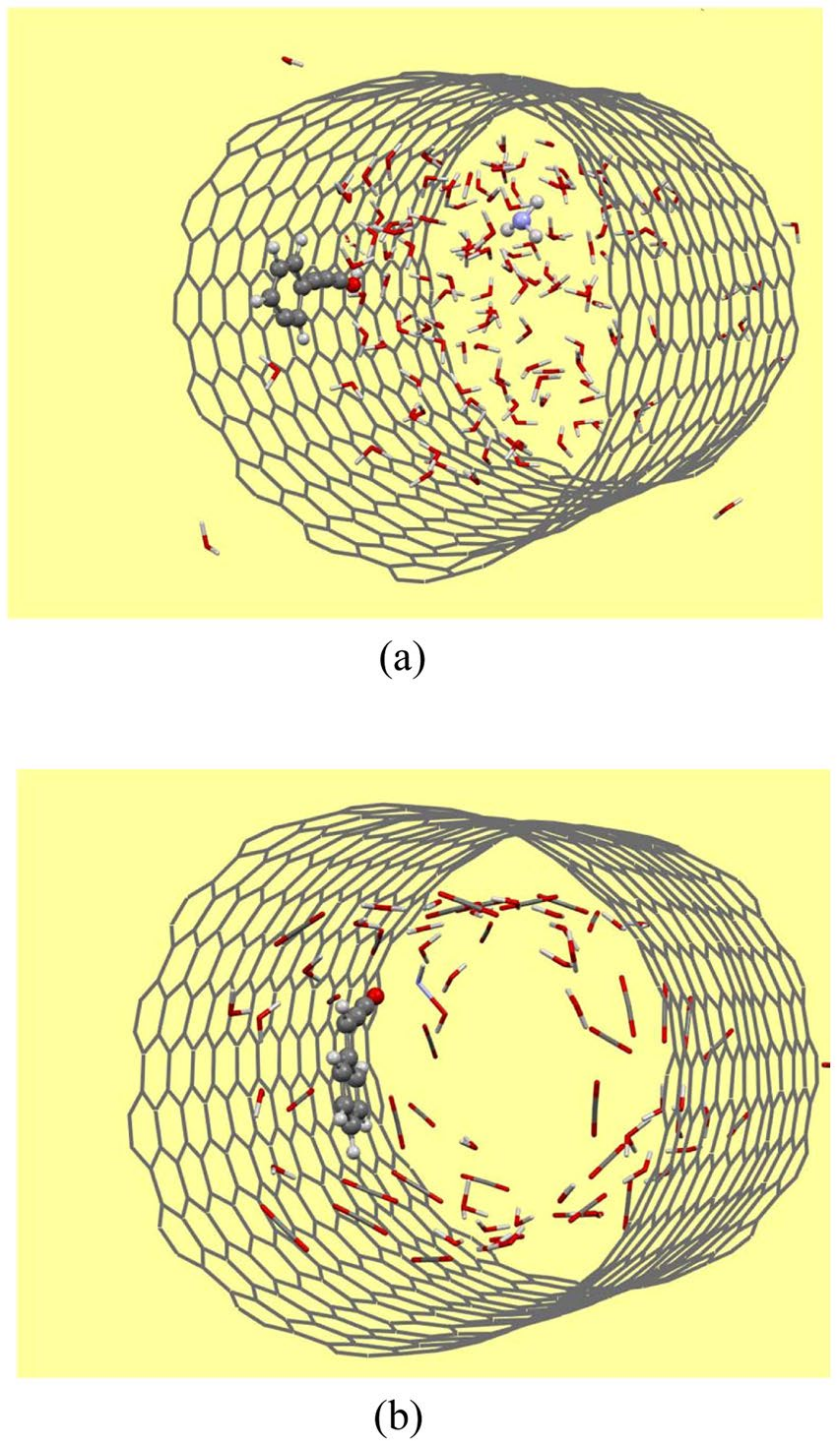
Systems containing the phenylalanine and (**a**) pure water and (**b**) binary mixture of water and 55% CO_2_ within the C-passive (15, 15) CNT at 200 ps.

Recently, Wang *et al*.^93^ demonstrated by using MD simulations that the edge of graphene hinders the movement of drug molecule hence it would be well-founded to evaluate the effect of CNT edge on the drug release. For this aim, carbon capped (15, 15) CNT (C-CNT) with two various solvent, pure water and carbonated water with 55% CO_2_, were considered and similar simulation procedure has been carried out for the systems under study. Our reactive MD simulation results showed that heating of considered systems up to 600 K did not cause the encapsulated drug to be released from the CNT. The snapshot of Phen/water@C-CNT (15, 15) and Phen/water-CO_2_@C**-**CNT (15, 15) systems at 400 K (~150 ps) is depicted in Fig. 3. As a result, CNT edge could not affect the movement of incorporated drug outward the tube and improve the drug release mechanism. Therefore, boiling of water molecules confined within nanotubes could not be an appropriate technique for drug release.

### Mechanism of release of drug and water molecules from the CNT

**Figure 4:**
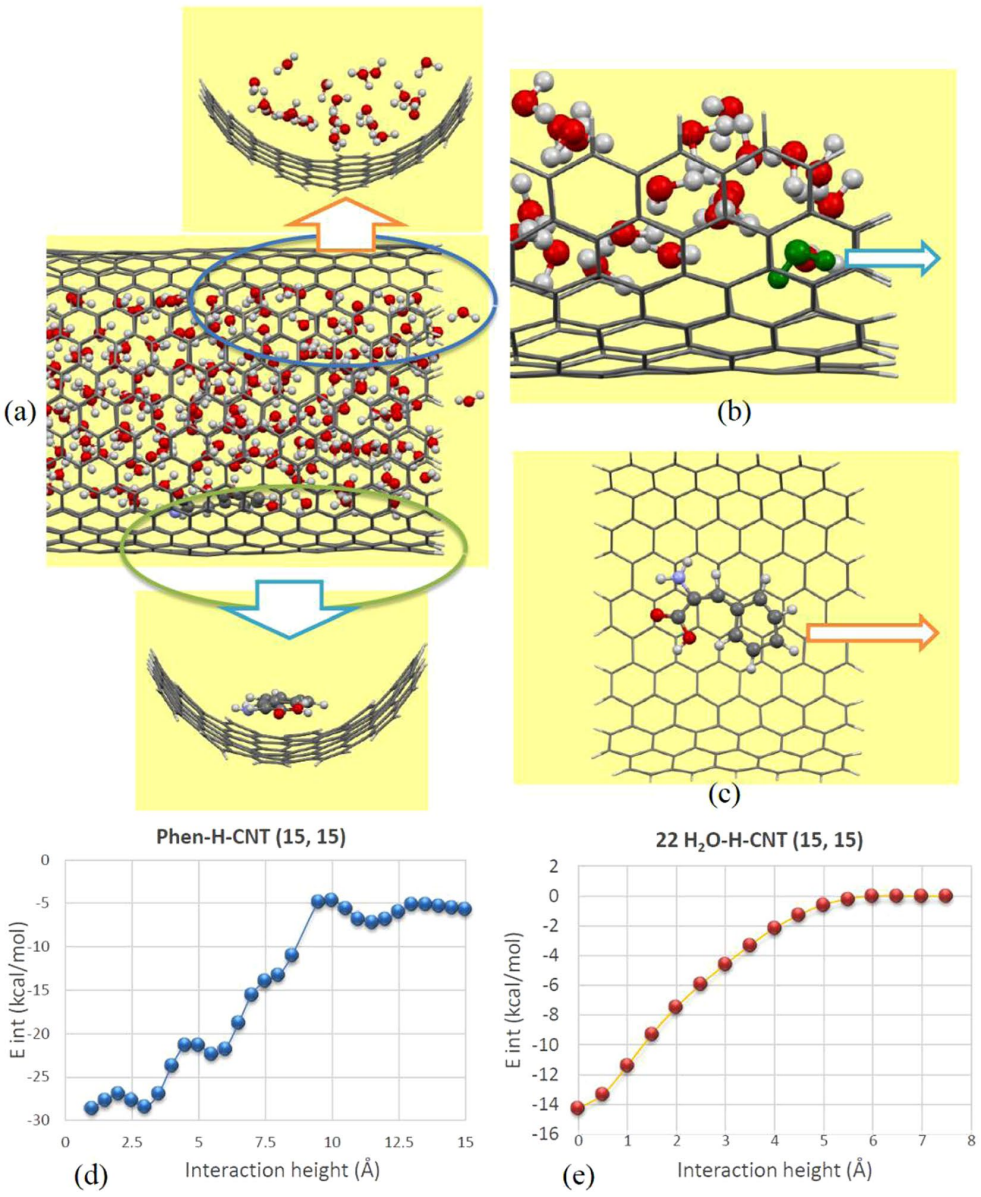
Schematic representation of (**a**) picked up structures of phenylalanine and water molecules interacting with (15, 15) CNT and scheme of (**b**) phenylalanine and (**c**) water (green colored molecule) molecules released from the CNT. Plots of calculated interaction energies, *E*_int_, versus interaction height for (**d**) phenylalanine and (**e**) water molecules released from CNT (15, 15).

To gain further insight into the mechanism of release of drug and water molecules from the CNTs the binding nature of phenylalanine and water molecule interacting with the sidewall of CNTs has been performed by using the *ab initio* calculations at the DFT level. The accuracy of our implemented DFT method (DFT-revPBE-D3) for similar systems has been validated elsewhere^85, 89, 92^. Because of computational restrictions, the selected system for the DFT calculations was reduced to a part of the CNT as represented in Fig. 4. As mentioned in the first section (Refs 52, 60 and 61), the structure and dynamics of water molecules confined within CNTs suffered significant changes compared to the bulk phase and hence the interaction nature of optimized system by MD simulation must be considered. Therefore, to simulate a realistic system we have picked up a section of system consisting of water molecules (22H_2_O molecules)/phenylalanine and a piece of nanotube adjacent to the adsorbed molecules which had been equilibrated at room temperature. Since the considered systems have been optimized by MD simulation thus we only performed single point energy calculations and then determined the interaction energies of systems under investigation by using the following relation:

2 $${E}_{{\rm{int}}}=E(\mathrm{Mol}\mbox{-}\mathrm{CNT})-[E({\rm{Mol}})+E({\rm{CNT}})]$$

where *E* (Mol-CNT) is the total energy of the whole system while *E*(Mol) and *E*(CNT) correspond to the total energies of water/phenylalanine molecule (Mol) and CNT, respectively.

Our first principles results, for the H-passive CNT, indicate that phenylalanine bound stronger to the interior sidewall of nanotube than the confined water molecules. The calculated interaction energies, *E*_int_, for the encapsulated phenylalanine molecule is about −1.239 eV (−28.58 kcal/mol) while the estimated *E*_int_ for water molecules is about −0.619 eV (−14.27 kcal/mol).

We have further evaluated the release of encapsulated molecules from the nanotube. For this propose we moved the phenylalanine and one H_2_O molecule toward the opening of CNT as represented in Fig. 4 and calculated the barrier energies of released molecules. Note that the position of water/phenylalanine has been fixed during the movement steps. For the water-CNT system we moved one of the water molecule which is positioned between nanotube sidewall and other water molecules to rationally account the attraction forces experienced by escaping water molecule from the tube cavity. Note that we considered a total number of 30/15 points for moved phenylalanine/water molecule along the tube axis with a spacing of 0.5 Å between points. The single point energy was then calculated for the whole system. The interaction energies between diffusing molecules and the nanotube sidewall as a function of interaction height was estimated and depicted in Fig. 4 for phenylalanine and water molecule. From the obtained values we can then determine the barrier energy of the released molecules from the nanotube (*E*_barrier_ = *E*_int-maximum_ − *E*_int-minimum_). As it can be found from the barrier energy values, the confined water molecule with barrier energy of about 0.617 eV (14.23 kcal/mol) could be released easier than the phenylalanine molecule with *E*_bar_ = 0.997 eV (22.99 kcal/mol). As a result, the strong binding of phenylalanine to the nanotube as well as significantly high barrier energy hinder drug molecule to leave the nanotube cavity. This finding confirms our MD simulation results which have demonstrated that water molecules could be released from the nanotube while the drug molecule prefers to be attached to the interior sidewall of the CNT.

For comparison, the effect of CNT edge has been also evaluated and the DFT results showed that C-passive edge could not improve the drug release process and encapsulated drug still encountered to a high barrier energy for the release. The calculated interaction energy and barrier energy for encapsulated drug into the C-passive CNT (15, 15) were determined to be −1.127 eV (−26.0 kcal/mol) and 0.907 eV (20.91 kcal/mol), respectively.

### Benchmark calculation

**Figure 5:**
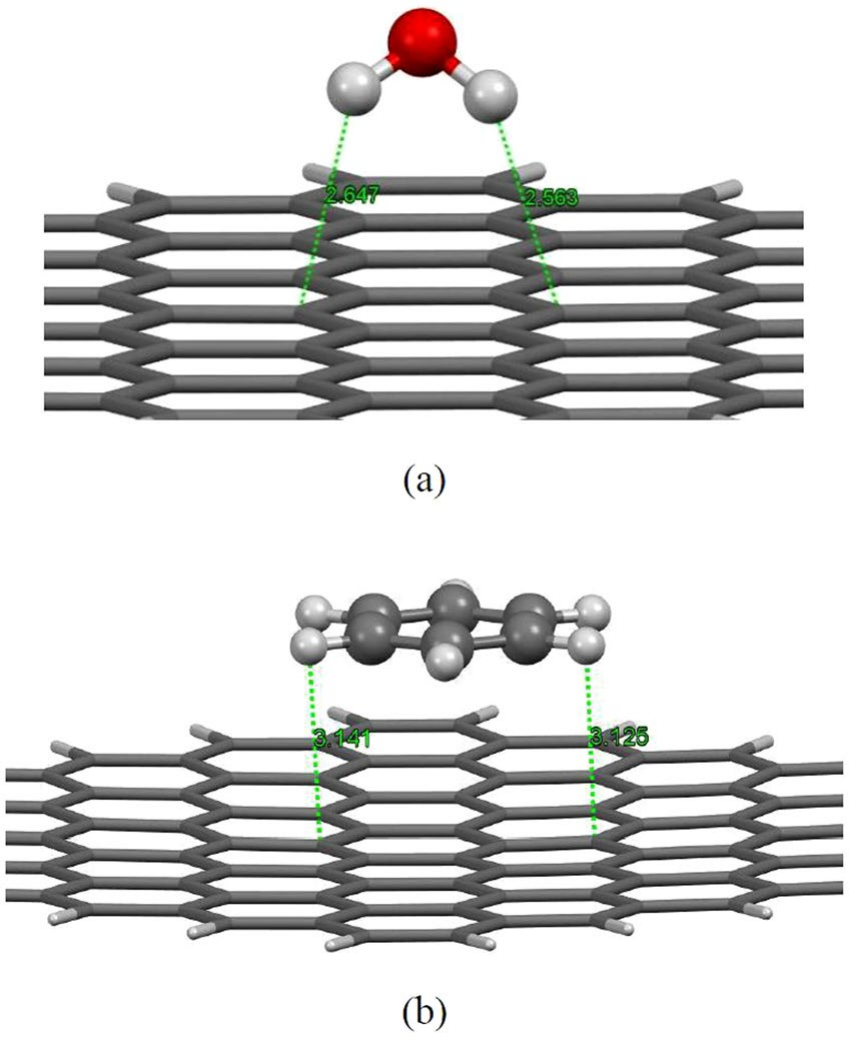
Optimized structures with DFT**-**revPBE-D3/SVP method for (**a**) H_2_O and (**b**) benzene molecules adsorbed onto graphene flake (All distances are in Å).

Finally to evaluate the validity of our DFT-revPBE-D3 calculation results we have compared this employed method with the existing research results in the literatures for similar systems. We first investigate the interaction of H_2_O molecule with graphene surface which has been studied by high level quantum mechanics methods at MP2 and CCSD level of theory. Following this aim, we have considered a hexagonal model consisting of 96 carbon atoms saturated with H atoms at the edge to demonstrate suitable model for the graphene sheet (see Fig. 5). The considered molecules were optimized separately and then fully structural optimization procedure was carried out for the combined systems. In order to evaluate the interaction between H_2_O molecule interacting with graphene surface, we eliminated the basis set superposition errors (BSSE) using the full counterpoise correction method^94^ for the optimized H_2_O/graphene system. This correction carried out by using ‘ghost’ atoms according to the following equation:

3 $${E}_{{\rm{ads}}}=E({{\rm{H}}}_{2}O\mbox{-}\mathrm{graph})-[{E({\rm{H}}}_{2}{{\rm{O}}}_{(\mathrm{ghost})}\mbox{-}graph)+E({{\rm{H}}}_{2}{O\mbox{-}\mathrm{graph}}_{(\mathrm{ghost})})]$$

where the “ghost” molecule corresponds to additional basis wave functions centered at the situation of H_2_O or graphene, but without any atomic potential. We used the TZVP(-f) basis set for the total energy (*E*_tot_) calculation. Our DFT-revPBE-D3 results showed an interaction energy of −0.127 eV (−2.929 kcal/mol) with average equilibrium distance (distance between H atom from H_2_O and C atom of graphene) of about 2.605 Å which are in good agreement with high-level quantum chemistry based calculations at the MP2 as well as CCSD(T) level of theory^95, 96^. The reported interaction energy and equilibrium distance between interacting molecules at the CCSD(T) level were determined to be −0.135 eV and 2.69 Å^95^, respectively, while MP2 method^96^ estimated −0.103 eV and 2.70 Å for the interaction energy and equilibrium distance, respectively.

To evaluate the accuracy of our DFT calculations for binding nature of phenylalanine with CNT surface we have considered the interaction of benzene molecule adsorbed onto the graphene surface (see Fig. 5) which its experimental data is available. Our DFT-revPBE-D3 calculation with TZVP(-f) basis set showed an interaction energy of about −0.56 eV (−12.88 kcal/mol) which agrees well with the experiment value of −13.6 kcal/mol^97^. These reasonable agreements between our DFT calculation results and experiment/high level quantum chemistry methods for water and benzene molecules interacting with graphene surface offer legal warranty of calculations accuracy for systems under study in the present work.

## Conclusions

The present research results from the reactive MD simulations can be used to explain the important physical features in the drug release process for confined water molecules within CNTs. One factor is that ultralow water-carbon friction causes the fast water transport into the nanotubes. Boiling of water molecules confined within the CNTs, on the other hand, causes to rise the inside pressure and release water molecules from the CNT. Meanwhile, it was expected that water release pushes the encapsulated drug molecule out of the nanotubes and hence an efficient drug release might be achieved. Another important factor is noncovalent π-π interaction between the aromatic ring of drug and the nanotube sidewall accompanied with the charge-transfer properties which dominant the binding strength of the interacting systems. All these factors which seem to be crucial for drug release by boiling of water molecules have been considered within the state-of-the-art ReaxFF potential in the present work. We modeled a phenylalanine amino acid embedded in water molecules as well as H_2_O/CO_2_ mixtures as too low boiling temperature solvent confined within various types of CNTs.

Our reactive MD simulation results which carried out up to 750 K demonstrated that encapsulated amino acid could not be released from the nanotubes by water/carbonated water boiling process. This observation can be attributed to the strong physisorption due to π-stacking interaction between the aromatic rings of phenylalanine and interior sidewall of CNTs. The equilibrium distance of the encapsulated phenylalanine from the sidewall of the CNTs was estimated to be about 3.0 Å. It was surprisingly observed that the encapsulated amino acid was decomposed when the temperature increased for some CNT systems and/or H_2_O/CO_2_ mixtures. Furthermore, the influence of CNT edge on the drug release has also been investigated and the MD simulation results revealed that C-passive CNTs have similar behavior with H-passive counterparts and phenylalanine could not get released from the nanotube at desirable temperature. This observation implies that boiling of water molecules confined within the CNTs could not be a suitable technique for efficient drug release.

We performed quantum mechanics calculations at the DFT level to explore the interaction nature of water and phenylalanine molecules confined within the CNT. Our DFT calculations showed that phenylalanine tend to bound stronger to the interior sidewall of CNT than the water molecules. The mechanism of release of water and phenylalanine molecules from the CNT has also been investigated. The calculated barrier energies for released molecules revealed also that barrier energy for phenylalanine was significantly higher than H_2_O molecule. These lead to harder release of phenylalanine from CNTs in comparison with confined water molecules as observed by MD simulation. The same result was also found for the C-passive CNT with rather similar values for the interaction and barrier energies for the encapsulated phenylalanine into the CNT. Our DFT calculations on the interaction energies of phenylalanine and H_2_O molecules confined/released within/from the nanotube successfully confirmed the MD simulation findings with ReaxFF potential. Meanwhile, the accuracy of DFT-revPBE-D3 calculations has been validated by experiment as well as high level quantum mechanics results at the MP2/CCSD level for similar systems. Overall, our semi-quantum MD simulation and DFT calculations findings give molecular origin insight into the structure and behaviors of encapsulated molecules under nanoscale confinement, and provide what to our knowledge are innovative evidences for the development of nanoscale drug delivery.

## Electronic supplementary material

Electronic Supplementary Information (ESI)
