# Supplementary material for: Molecular origin of drug release by water boiling inside carbon nanotubes from reactive molecular dynamics simulation and DFT perspectives
Source: Sci Rep. 2017 Jul 5;7:4669. doi: 10.1038/s41598-017-04981-2 (PMC5498575; doi:10.1038/s41598-017-04981-2)
Supplement: Supplementary file 1 — Electronic Supplementary Information (ESI) [file 41598_2017_4981_MOESM1_ESM.pdf]

Supporting Information

Molecular origin of drug release by water boiling inside carbon nanotubes from reactive molecular dynamics simulation and DFT perspectives

M. Darvish Ganji [a, *], Sh. Mirzaei [b] and Z. Dalirandeh [c]

[a] Department of Nanochemistry, Faculty of Pharmaceutical Chemistry, Pharmaceutical Sciences Branch, Islamic Azad University, Tehran - Iran (IAUPS)

[b] Department of Chemistry, Qaemshahr Branch, Islamic Azad University, Qaemshahr, Iran

[c] Young Researchers and Elite Club, Central Tehran Branch, Islamic Azad University, Tehran, Iran.

Corresponding Authors: [*]E-mail address: ganji_md@yahoo.com (M.D. Ganji).

Captions to Tables and Figures:

Table S1. The position details of the phenylalanine molecule inside the CNTs at 50 ps. (all units are in (Å)).

Table S2. The position details of encapsulated phenylalanine molecule inside the CNTs for CO_2 percentage of (a) 20%, (b) 40% and (c) 55% at 350 K and 50 ps (all units are in (Å)).

Fig. S1. Schematic representation of (a) (21, 9) CNT, (b) (26, 0) CNT and (c) (15, 15) CNT.

Fig. S2. Systems containing the phenylalanine and binary mixture of water and 20% CO_2 within the (a) (15, 15) CNT (b), (21, 9) CNT and (c) (26, 0) CNT at 50 ps.

Table S1

CNT	(15, 15)	(21, 9)	(26, 0)
d_{CNT}	21.145	20.401	16.609
L_{CNT}	29.291	29.768	28.062
L_{phe-F}	12.210	25.119	23.815
L_{phe-E}	14.618	6.071	4.203
d_{phe-W1}	15.968	17.515	16.513
d_{phe-W2}	3.622	3.588	3.626

d_{CNT}: CNT diameter
L_{CNT}: CNT length
L_{phe-F}: distance from the phenylalanine molecule to CNT fore
L_{phe-E}: distance from the phenylalanine molecule to CNT end
d_{phe-W1}: distance from the phenylalanine molecule to wall 1
d_{phe-W2}: distance from the phenylalanine molecule to wall 2

Table S2

(a)

CNTs	(15, 15)	(26, 0)	(21, 9)
d_{1CNT}	20.207	19.978	19.153
d_{2CNT2}	20.743	20.594	20.022
L_{CNT}	29.370	28.287	31.366
$L_{phe\text{-}F}$	19.576	12.615	16.285
$L_{phe\text{-}E}$	9.797	15.458	15.082
$d_{phe\text{-}W1}$	17.538	17.794	16.507
$d_{phe\text{-}W2}$	2.980	3.335	3.890
$d_{phe\text{-}N}$	-	Nitrogen is out of the nanotube	-
$d_{N\text{-}W1}$	-		-
$d_{N\text{-}W2}$	-		-

(b)

CNTs	(15,15)	(26,0)	(21,9)
d_{1CNT}	19.626	18.963	19.288
d_{2CNT2}	20.920	18.835	20.089
L_{CNT}	29.416	28.271	31.505
$L_{phe\text{-}F}$	7.357	7.119	7.400
$L_{phe\text{-}E}$	22.062	21.179	24.122
$d_{phe\text{-}W1}$	17.256	16.631	16.917
$d_{phe\text{-}W2}$	3.292	3.340	3.312
$d_{phe\text{-}N}$	17.116	in range with Benzene ring	-
$d_{N\text{-}W1}$	7.302	7.068	-
$d_{N\text{-}W2}$	21.930	21.282	-

(c)

CNTs	(15,15)	(26,0)	(21,9)
d_{1CNT}	19.591	19.965	20.843
d_{2CNT2}	20.732	18.491	19.879
L_{CNT}	29.367	28.268	31.272
$L_{phe\text{-}F}$	13.590	21.182	5.164
$L_{phe\text{-}E}$	15.924	7.097	25.858
$d_{phe\text{-}W1}$	17.239	16.722	17.52
$d_{phe\text{-}W2}$	3.495	3.364	3.837
$d_{phe\text{-}N}$	-	in range with Benzene ring	17.670
$d_{N\text{-}W1}$	-	10.406	4.453
$d_{N\text{-}W2}$	-	4.680	15.029

(a)

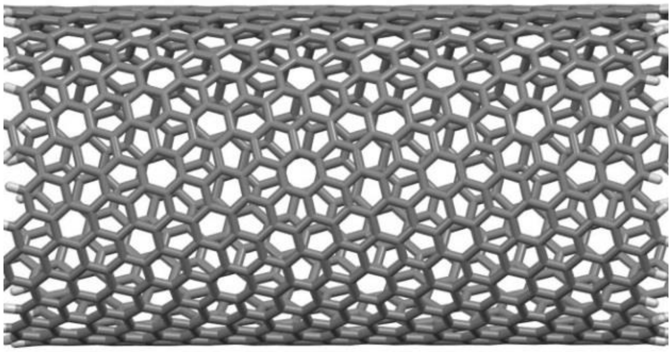

(b)

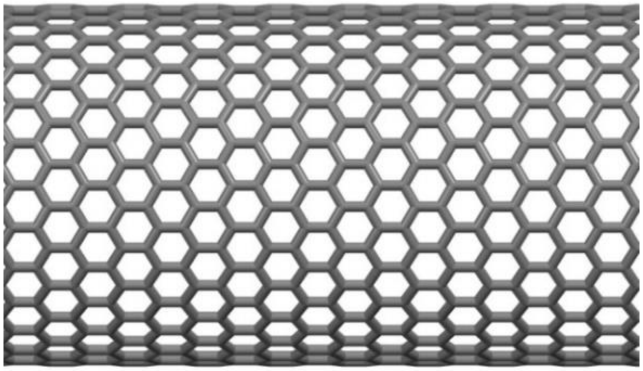

(c)

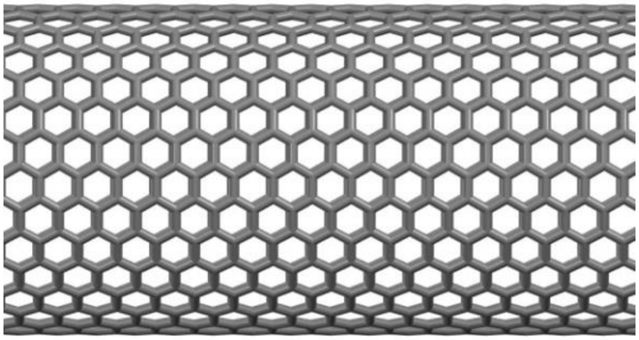

Fig. S1

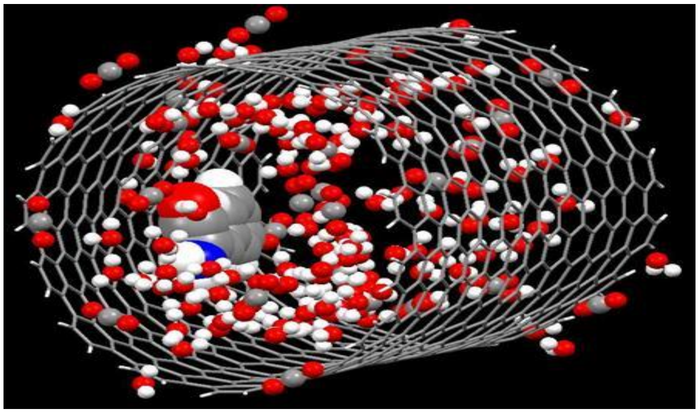

(a)

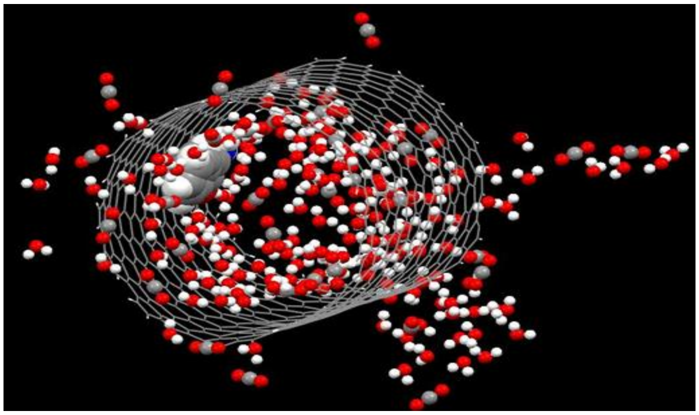

(b)

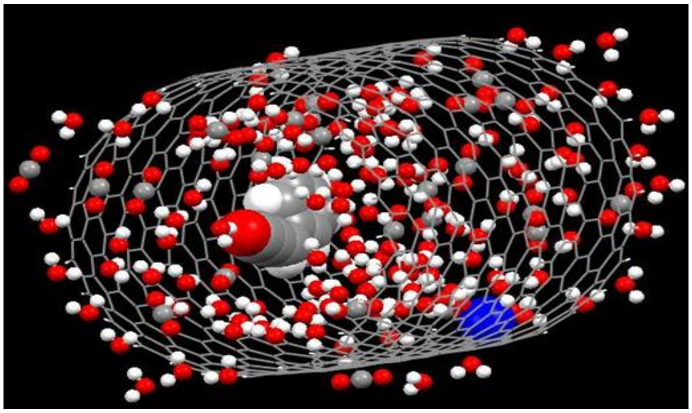

(c)

Fig. S2
